# Abnormal metabolic connectivity in default mode network of right temporal lobe epilepsy

**DOI:** 10.3389/fnins.2023.1011283

**Published:** 2023-03-23

**Authors:** Xiaoyang Wang, Dandan Lin, Chunlei Zhao, Hui Li, Liyuan Fu, Zhifeng Huang, Shangwen Xu

**Affiliations:** ^1^Department of Medical Imaging, The 900th Hospital of Joint Logistic Support Force, PLA, Fuzhou, Fujian, China; ^2^Department of Medical Imaging, Affiliated Dongfang Hospital, Xiamen University, Fuzhou, Fujian, China; ^3^Department of Clinical Medicine, Fujian Health College, Fuzhou, Fujian, China

**Keywords:** TLE, 18F-FDG PET, metabolic connectivity, default mode network, graph theory

## Abstract

**Aims:**

Temporal lobe epilepsy (TLE) is a common neurological disorder associated with the dysfunction of the default mode network (DMN). Metabolic connectivity measured by 18F-fluorodeoxyglucose Positron Emission Computed Tomography (18F-FDG PET) has been widely used to assess cumulative energy consumption and provide valuable insights into the pathophysiology of TLE. However, the metabolic connectivity mechanism of DMN in TLE is far from fully elucidated. The present study investigated the metabolic connectivity mechanism of DMN in TLE using 18F-FDG PET.

**Method:**

Participants included 40 TLE patients and 41 health controls (HC) who were age- and gender-matched. A weighted undirected metabolic network of each group was constructed based on 14 primary volumes of interest (VOIs) in the DMN, in which Pearson’s correlation coefficients between each pair-wise of the VOIs were calculated in an inter-subject manner. Graph theoretic analysis was then performed to analyze both global (global efficiency and the characteristic path length) and regional (nodal efficiency and degree centrality) network properties.

**Results:**

Metabolic connectivity in DMN showed that regionally networks changed in the TLE group, including bilateral posterior cingulate gyrus, right inferior parietal gyrus, right angular gyrus, and left precuneus. Besides, significantly decreased (*P* < 0.05, FDR corrected) metabolic connections of DMN in the TLE group were revealed, containing bilateral hippocampus, bilateral posterior cingulate gyrus, bilateral angular gyrus, right medial of superior frontal gyrus, and left inferior parietal gyrus.

**Conclusion:**

Taken together, the present study demonstrated the abnormal metabolic connectivity in DMN of TLE, which might provide further insights into the understanding the dysfunction mechanism and promote the treatment for TLE patients.

## Introduction

Temporal lobe epilepsy (TLE) is the most common intractable epilepsy in adults, accounting for 30–35% of the 50 million worldwide affected by epilepsy (Gutierrez-Barragan et al., 2019; Wang et al., 2019). TLE is characterized by epileptic focus in hippocampus, amygdala or entorhinal cortex (Trimmel et al., 2018; Ito et al., 2021). Increasing studies have demonstrated that abnormal dysfunction caused by TLE was involved in extra-temporal regions rather than limited in the temporal lobe (Zhou et al., 2019). Furthermore, a large number of studies argued that TLE is not only a focal disease but also associated with brain network dysfunction containing the intra- and extra-temporal regions, and the extratemporal metabolic abnormalities could affect the long-term surgical prognosis (Sager et al., 2011; Chassoux et al., 2016, 2017; Liu et al., 2016; Trimmel et al., 2018; Laurent et al., 2020; Tang et al., 2020). Specifically, studies have shown that deficits in metabolic network which is essential for the normal functioning of the brain, including memory, emotion, and perception, may contribute to the development and progression of TLE (Laurent et al., 2020; Cho et al., 2022). Thus, the research on the dysfunctional brain network in TLE would contribute to the understanding of the pathogenesis of epilepsy, which would be conducive to the clinical diagnosis and treatment for epilepsy.

Default mode network (DMN) is an intrinsic brain network which participates in spontaneous functional activity in the resting state and becomes less active during attention-demanding tasks (Raichle et al., 2001; Simoni et al., 2016; Zhang and Volkow, 2019). DMN is involved in a variety of cognitive functions, including self-awareness, episodic memory retrieval, and conceptual processing, it is thought to be important for consciousness and the self-monitoring of internal states (Lu et al., 2012; Zhang and Volkow, 2019; Cui et al., 2022). Besides, dysfunction of DMN has been implicated a significant characteristic in a number of psychiatric disorders, including Alzheimer’s disease (Chiesa et al., 2019; Lu et al., 2019), Parkinson’s disease (Hou et al., 2017; Franciotti et al., 2019; Zhong et al., 2019) and TLE (Haneef et al., 2012; Zhou et al., 2019). Meanwhile, it has been demonstrated that executive deficits in TLE were associated with abnormal connectivity in DMN (Zhou et al., 2019). Specially, previous studies found that functional connectivity alterations in DMN in TLE patients, specifically decreased connectivity between both medial temporal lobes and the posterior part of the DMN and increased interhemispheric anterior-posterior connectivity (McCormick et al., 2014). Partial epilepsy patients showed significantly decreased functional connectivity in DMN regions such as right uncus, left inferior parietal lobule, left supramarginal gyrus, left uncus, left parahippocampal gyrus, and left superior temporal gyrus, in epilepsy patients, compared to healthy controls (Hu et al., 2017).

With the development of neuroimage techniques, the functional, structural, and metabolic connectivity in DMN could be well explored by functional magnetic imaging (fMRI), diffusion tensor imaging (DTI), and positron emission tomography (PET), respectively (Di et al., 2012; Robinson et al., 2016; Zhang and Volkow, 2019). Previous studies have found that both the functional and structural connectivity of DMN in TLE was abnormal (Xue et al., 2013; Hsiao et al., 2015; Zhou et al., 2019). Different from the correlation of rapid temporal fluctuations estimated by fMRI-based functional connectivity and the fiber connection measured by DTI-based structural connectivity, metabolic connectivity assessed by 18F-FDG PET could reflect cumulative energy consumption in the resting state. Compared with functional connectivity, metabolic connectivity showed a higher signal-to-noise ratio with an assumed steady state of neuronal activity during the recording interval and an inherently less dependent neurovascular coupling (Sun et al., 2019). Therefore, metabolic connectivity is a relatively stable indicator to reflect the physiological and pathological levels of the brain and has been widely used in neurological and psychiatric diseases (Di et al., 2012; Titov et al., 2017; Yakushev et al., 2017; Liang et al., 2018; Verger et al., 2020; Jamadar et al., 2021). However, few studies have focused on the metabolic connectivity in DMN of TLE.

In the present study, 18F-FDG PET was applied to assess the metabolic connectivity of DMN for both TLE and healthy groups to research the metabolic connectivity of DMN in TLE. Besides, the topological properties were further estimated to explore the features of DMN metabolic connectivity using the grape theory analysis method.

## Materials and methods

### Participants

All participants were recruited from the epilepsy clinic of the 900th Hospital of Joint Logistic Support Force, PLA. A total of 40 patients with TLE and 41 healthy controls who were age- and gender-matched were enrolled in the present study. All participants were right-handed and underwent comprehensive standard clinical assessments, including medical history, neurological examinations, EEG recording, MRI, and PET scanning. All patients who underwent diagnosis of TLE were performed by at least two experienced neurologists based on the diagnostic criteria of the International League Against Epilepsy. Specifically, the TLE patients met at least two of the following three inclusion criteria: (1) typical symptoms of epileptic seizures indicated that the epileptogenic lesion was located in the right-side temporal lobe; (2) EEG recordings showed epileptic discharges from the right-side temporal lobe; and (3) brain magnetic resonance imaging scanning revealed atrophy or sclerosis of the right-side hippocampus, but no other specific abnormalities in the brain. All the healthy controls in this study were recruited from the people who had a physical examinations at the 900th Hospital of Joint Logistic Support Force, PLA in recent years. All participants were excluded if other cerebral structural abnormalities, such as infarction, tumor or demyelination, severe physical or mental diseases and the epileptogenic lesion were not in the temporal lobe. Patients who could not cooperate during the examinations were also excluded. The Medical Research Ethics Committee approved this study of the 900th Hospital of Joint Logistic Support Force, PLA, and informed consent from each participant was obtained prior to the study (2019-005).

### PET scans

18F-FDG was prepared at the PET center of the 900th Hospital of Joint Logistic Support Force, PLA. The PET scans operate in strict accordance with the guidelines. Participants were fasted and water is forbidden for 12 h before the FDG PET was performed, and avoided taking drugs that affected glucose metabolism. All participants tried to relax during the scan. During the whole process of PET imaging, a neurologist observed the subjects through the window. When the participants felt uncomfortable, they could press the alarm and the scanner would immediately stop the experiment. 18F-FDG PET images were acquired on a General Discovery LS PET scanner, of which the radial spatial resolution is 4.25 mm full-width at half-maximum (FWHM) at the center of the FOV. The brain of each participant was centered in the FOV to perform a static acquisition of 15 min at 50 min after intravenous injection of 0.15 mCi/kg FDG. Images were reconstructed on a 128 × 128 × 35 matrix, where the voxel size equals 1.95 × 1.95 × 4.25 mm. All scans were saved in Analyze format.

### PET data preprocessing

All preprocessing was performed using Statistical Parametric Mapping12 (SPM12^1^) (Ashburner, 2012). Firstly, all PET images were spatially normalized into the Montreal Neurological Institute (MNI) brain space. Then all the spatial normalized PET images were smoothed using a Gaussian kernel of 8 mm FWHM. For scaling voxel intensities, the voxel counts were normalized by the mean intensity of the brain in each participant’s PET image.

### Construction of connectivity matrix

To generate a brain network, 14 primary volumes of interest (VOIs) (Table 1) in the DMN were selected as nodes according to the automated anatomical labeling (AAL) atlas. Intensity-normalized FDG uptake in VOIs of each subject was obtained, and then Pearson’s correlation coefficients between each pair-wise of the VOIs were calculated in an inter-subject manner. A weighted undirected network matrix (14 × 14) was constructed for both the TLE and health control (HC) groups, in which the strength of each the connection was defined as correlation coefficient (Kaiser, 2011; Titov et al., 2017; Yakushev et al., 2017).

**TABLE 1:** List of volumes of interest in DMN.

Number	Region name	Abbreviation	MNI coordinate		
			***x***	***y***	***z***
1	Left superior frontal gyrus, medial	SFGmed.L	−6	49	31
2	Left superior frontal gyrus, medial orbital	ORBsupmed.L	−6	54	−7
3	Left posterior cingulate gyrus	PCG.L	−6	−43	25
4	Left hippocampus	HIP.L	−26	−21	−10
5	Left inferior parietal gyrus	IPL.L	−44	−46	47
6	Left angular gyrus	ANG.L	−45	−61	36
7	Left precuneus	PCUN.L	−8	−56	48
8	Right superior frontal gyrus, medial	SFGmed.R	8	51	30
9	Right superior frontal gyrus, medial orbital	ORBsupmed.R	7	52	−7
10	Right posterior cingulate gyrus	PCG.R	6	−42	22
11	Right hippocampus	HIP.R	28	−20	−10
12	Right inferior parietal gyrus	IPL.R	45	−46	50
13	Right angular gyrus	ANG.R	45	−60	39
14	Right precuneus	PCUN.R	9	−56	44

Coordinates (*x*, *y*, and *z*) refer to the standard MNI space, and each coordinate indicates the central voxel location within each brain region.

### Graph theory analysis

Based on the weighted undirected network, the distance matrix of TLE and HC was generated. Graph theoretical approaches using Brain Connectivity Toolbox^2^ (Rubinov and Sporns, 2010) and graph theoretical network analysis (GRETNA^3^) (Wang et al., 2015) were applied to characterize the functional connectivity pattern of TLE and HC. The global efficiency and the characteristic path length were used to assess the global network properties. Nodal efficiency and degree centrality were evaluated for each node to assess regional network properties.

To determine the significant statistical differences in network parameters between the TLE and HC groups, a permutation test with 10,000 times was performed. *P* < 0.05 was considered statistically significant.

### Statistical analysis

The permutation test was performed to assess the statistical comparison of the network matrix between TLE and HC. The network matrix of TLE and HC was transformed to the *Z* scores using Fisher transformation. PET images were randomly permuted 10,000 times into pseudo-random groups and the network matrix was calculated. Then, all the network matrix was transformed using Fisher transformation. Type I error was determined by the comparison between the observed *Z* score for each connection and *Z* score distribution from the permuted data. False discovery rate (FDR) was applied to correct for multiple comparisons at a threshold of *q* < 0.05.

An independent two-sample *t*-test was performed to examine the age between patients and healthy control groups. A Chi-square test was performed to compare the gender distribution between patients and healthy control groups. These clinical data were analyzed in SPSS20. *P* < 0.05 was considered statistically significant.

## Results

### Demographic characteristics evaluation

There was no significant difference in age and gender between patients with TLE and HC. The detailed clinical data of the participants was described in Table 2.

**TABLE 2:** Demographic and clinical characteristics of all participants.

	TLE	HC	*P*
Participants (number)	40	41	–
Gender (M/F)	23/17	25/16	0.75
Age (years)	34.93 ± 7.89	36.46 ± 7.42	0.37
Age of symptoms onset (years)	22.35 ± 10.57	–	–
Epilepsy duration (years)	12.42 ± 8.20	–	–
Duration of each symptom (minutes)	2.36 ± 2.02	–	–

Results are displayed as mean ± SD.

### Characteristic global and regional graph theory measures in the TLE group

**FIGURE 1:**
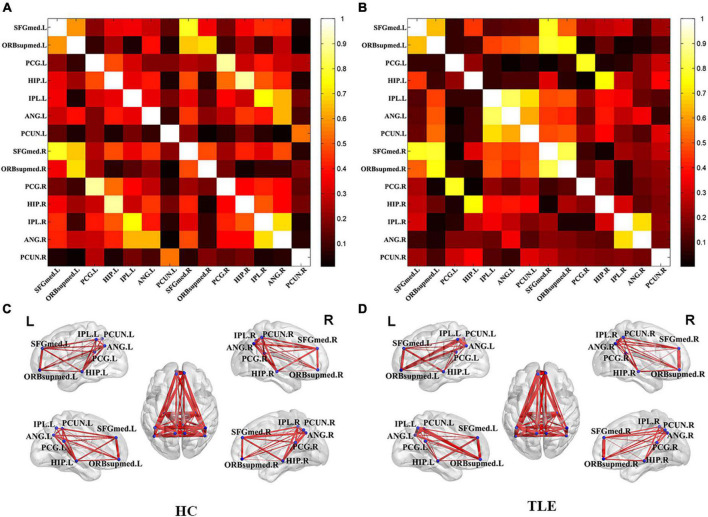
Metabolic correlation network of the TLE and HC groups. The left panel was the connectivity matrix **(A)** and connectivity graph **(C)** of the HC group. The left panel was the connectivity matrix **(B)** and connectivity graph **(D)** of the TLE group. The nodes of VOIs by blue dots and connection strength is represented by the thickness of each line connecting two nodes. TLE, temporal lobe epilepsy; HC, healthy control.

To further study the brain function of the TLE, metabolic connectivity was conducted in an inter-subject manner, which was supposed to reflect inter-regional covariance patterns of neuronal activities. The correlation matrix represented the connection strength of pair-wise VOI and the connectivity graph was constructed for the HC (Figure 1A) and TLE (Figure 1B) groups. To evaluate the connectivity of each group in visualization, connectivity graphs were shown for the HC (Figure 1C) and TLE (Figure 1D) groups.

**FIGURE 2:**
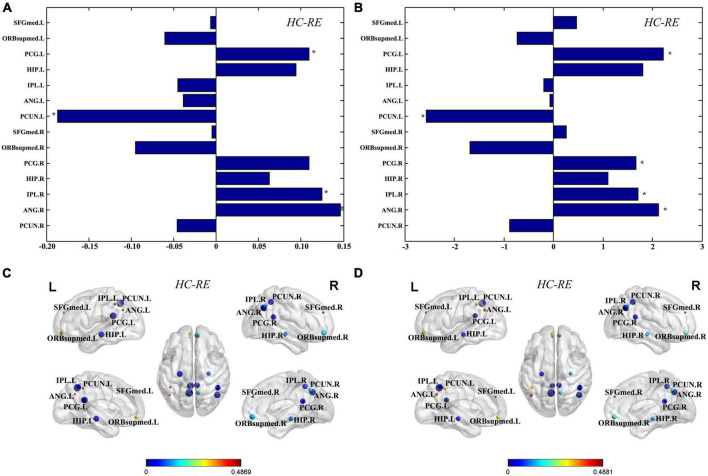
Regional graph theoretic measures. Differences of nodal efficiency **(A)** and degree centrality **(B)** for each node were evaluated between the TLE and HC group. The differences of nodes in nodal efficiency **(C)** and degree centrality **(D)** between the TLE and HC groups were displayed in the MNI space. The size of nodes represents difference between the TLE and HC groups, and the color of nodes and the color bar represent statistical significance. *Statistically significant (q < 0.05, FDR corrected). SFGmed, superior frontal gyrus, medial; ORBsupmed, superior frontal gyrus, medial orbital; PCG, posterior cingulate gyrus; HIP, inferior parietal gyrus; IPL, inferior parietal gyrus; ANG, angular gyrus; PCUN, precuneus; L, left; R, right.

Graph theory was used to further investigate the network parameters of TLE and HC groups. There were no significant differences in global properties including global efficiency and the path length between the TLE and HC groups. Compared to the HC group, the TLE group showed significantly decreased nodal efficiency in left PCG, right IPL, and right ANG, and significantly increased nodal efficiency in left PCUN (Figure 2A). In addition, compared with the HC group, degree centralities of bilateral PCG, right IPL, and right ANG in TLE group are significantly decreased, while the degree centrality of left PCUN in TLE group was significantly increased (Figure 2B). According to the anatomical localization and regional graph measures, the differences of nodal efficiency (Figure 2C) and degree centrality (Figure 2D) for each node were displayed.

### Disrupted metabolic connectivity in the TLE group

**FIGURE 3:**
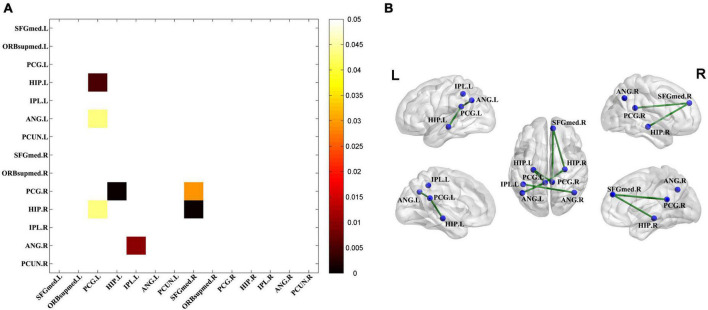
Significantly decreased metabolic connectivity in the TLE groups. **(A)** The seven significantly decreased metabolic connections in the TLE groups compared with the HC group (permutation test, *P* < 0.05 with FDR correction). Statistical significance of the reduction of metabolic connectivity in the TLE group was found and represented by lower triangular matrix. **(B)** The anatomical distribution of significantly different metabolic connections in pairwise VOIs between the TLE and HC groups.

Significantly decreased connectivity between pairwise VOIs was examined using permutation tests (Figure 3A) and exhibited by connectivity graph (Figure 3B). The seven significantly decreased metabolic connections were found as follows: HIP.L – PCG.L, ANG.L – PCG.L, HIP.R – PCG.L, PCG.R – HIP.L, ANG.R – IPL.L, PCG.R – SFGmed.R, and HIP.R – FGmed.R.

## Discussion

The present study applied a large-scale network analysis based on glucose metabolism as measured by 18F-FDG PET was applied to assess the metabolic connectivity in the DMN of patients with TLE. Graph-theoretic analysis of the metabolic connectivity in the DMN implied that regional networks changed in the TLE group. In addition, an abnormal metabolic connection in the DMN containing bilateral HIP, bilateral PCG, and bilateral ANG, right SFGmed, and left IPL was found in the TLE group.

Metabolic connectivity assessed by 18F-FDG PET is based on the coupling of neuronal activity and brain energy consumption during the steady resting state, which could provide new insight into the understanding of brain functional connectivity (Wang et al., 2015). Previous studies have proven that there existed a metabolic DMN *via* the region of interest-based correlation analysis on a series of 18F-FDG PET images (Di et al., 2012; Gao et al., 2018, 2022; Zhou et al., 2020; Li et al., 2021, 2022). The brain functional network generated by PET and fMRI measured different periods of participants: the fMRI measures real-time brain function activity during imaging, while 18F-FDG PET measures accumulation of FDG metabolism. Thus, DMN measured by FDG PET reflected energy consumption in participants the 50-min uptake period. Thus metabolic network analysis may provide a reliable access to study DMN. In addition, according to the previous studies on the metabolic networks, the number of subjects in the present study is appropriate for analyzing the metabolic connectivity *via* the inter-subject method (Sala et al., 2017). Based on the VOI correlation analysis of FDG-PET images in an inter-subject manner, the study found abnormal metabolic connectivity in DMN of TLE patients.

Graph theoretic theory analysis has been extensively used in the analysis of medical image data. A number of previous studies suggested that graph-theoretic analysis could be much more powerful than other methods applied to brain networks, especially in the analysis of large-scale brain networks. Global efficiency and characteristic path length are typically used to study the global properties of brain networks in neurological diseases (Chaitra et al., 2020). Global efficiency is associated with the capacity of the network in parallel information transferring between nodes *via* multiple series of edges, which reflects the network efficiency in transferring information (Zhou et al., 2022). Characteristic path length reflects the nodes that the information needs to cross to reach its final destination (Chaitra et al., 2020). The DMN is a high topological organization and low consumption. Previous studies based on fMRI reported that the global properties of DMN in TLE were the same as in HC (Zhou et al., 2019). Analysis of metabolic connectivity in DMN showed that there was no significant difference in the global properties of global efficiency and characteristic path length between the TLE and HC groups, which was consistent with the previous studies.

To further investigate the local properties for each node of metabolic DMN in TLE, the nodal efficiency and degree centrality were estimated in the present study. The local properties of most nodes were significantly decreased in the metabolic DMN, except the left PCUN. The present study found both the nodal efficiency and degree centrality of the right inferior parietal gyrus and right angular gyrus in DMN were significantly decreased in TLE, which was consistent with a previous study on TLE *via* resting-state fMRI (Wang et al., 2014; Bruner et al., 2017). Precuneus is involved in consciousness, episodic memory, visuospatial, and motor activity coordination, and several studies have found that the higher cognitive processing of precuneus is accompanied by higher metabolic rates and energy consumption (Bruner et al., 2017; Rousseau et al., 2019). In the present study, the increase of nodal efficiency and degree centrality in the left precuneus was found, which might act as a compensatory role for the disrupted metabolic connectivity in DMN of TLE. Besides, studies have proven that the posterior cingulate gyrus is the hub node in DMN, which is closely connected to other nodes to play an important role in regulating and transferring information within the network (Lu et al., 2012; Zhang and Volkow, 2019). It has been reported that disruptions of the hub nodes in DMN might underlie the pathophysiological mechanism of epilepsy in a resting-state fMRI study (Wang et al., 2017). As a component of the DMN and a part of the limbic system, the posterior cingulate gyrus plays an important role in visuospatial, memory, and movement functions, which involves processing the information of the spatial orientation and navigation. The abnormality of the posterior cingulate gyrus is believed to lead to the propagation of epileptic discharges (Enatsu et al., 2014).

Furthermore, reduced metabolic connectivity included bilateral posterior cingulate gyrus, bilateral hippocampus, bilateral angular gyrus, right medial superior frontal gyrus, and left inferior parietal gyrus in the DMN of the TLE group was found using the metabolic connectivity comparison. This finding has shown that the bilateral posterior cingulate gyrus plays the role of bridge to connect with the nodes in the contralateral hemisphere. Studies have found that the nodes in the abnormal metabolic network play essential roles in consciousness, visuospatial, learning and memory, and the control of motor activity (Lu et al., 2012; Wang et al., 2017; Zhang and Volkow, 2019). In addition, previous studies have shown that improvement of the abnormal brain network could promote the rehabilitation of the dysfunction after brain disorders (Liang et al., 2017; Allendorfer et al., 2019). The abnormal metabolic connection might provide insight into the treatment of TLE patients.

There were several limitations in this study. A certain network measurement may reflect a specific profile of the regional function. This study focused on the nodal efficiency and degree centrality, while other local network attributes such as nodal local efficiency and clustering coefficient were not fully studied. Although the nodal efficiency and degree centrality were the most commonly used local network attributes in neuroimaging studies, studying more local network attributes would provide more details about their effects on neuronal activity. This may be a promising direction in future studies.

## Conclusion

In conclusion, based on FDG-PET, the present study found an abnormal metabolic connection central at bilateral posterior cingulate gyrus in DMN of TLE, which would provide further insights into the understanding the dysfunction mechanism and promoting the treatment for TLE patients.

## Data availability statement

The raw data supporting the conclusions of this article will be made available by the authors, without undue reservation.

## Ethics statement

The Medical Research Ethics Committee approved this study of the 900th Hospital of Joint Logistic Support Force, PLA, and informed consent from each participant was obtained prior to the study (2019-005). The patients/participants provided their written informed consent to participate in this study. Written informed consent was obtained from the individual(s) for the publication of any potentially identifiable images or data included in this article.

## Author contributions

XW: writing—original draft, and writing—review and editing. DL: writing—original draft. CZ and HL: investigation. LF: software. ZH: validation. SX: conceptualization and project administration. All authors contributed to the article and approved the submitted version.

## Footnotes

1
https://www.fil.ion.ucl.ac.uk/spm/

2
https://sites.google.com/site/bctnet/

3
https://www.nitrc.org/projects/gretna
